# Quantification of abdominal aortic calcification using photon-counting CT angiography: an imaging biomarker for high-risk cardiovascular patients

**DOI:** 10.1007/s11547-025-01978-0

**Published:** 2025-03-28

**Authors:** Takashi Ota, Atsushi Nakamoto, Masatoshi Hori, Hideyuki Fukui, Hiromitsu Onishi, Mitsuaki Tatsumi, Noriyuki Tomiyama

**Affiliations:** 1 Department of Diagnostic and Interventional Radiology, Osaka University Graduate School of Medicine, D1, 2-2, Yamadaoka, Suita, Osaka 565-0871 Japan; 2 Department of Artificial Intelligence in Diagnostic Radiology, Osaka University Graduate School of Medicine, Suita, Japan; 3 Department of Medical Physics and Engineering, Osaka University Graduate School of Medicine, Suita, Japan

**Keywords:** Photon-counting CT, Abdominal atherosclerosis, Cardiovascular disease, CT angiography, Virtual non-calcium, Calcification volume

## Abstract

**Objectives:**

To evaluate abdominal aortic calcification parameters derived from 3D volumetric analysis using photon-counting CT (PCCT) angiography-based virtual non-calcium (VNCa) algorithm as an imaging biomarker for high-risk cardiovascular disease (CVD) patients.

**Methods:**

This retrospective study included patients who underwent abdominal PCCT angiography and non-contrast-enhanced chest CT (nCE-CCT, including CT scanners other than PCCT) between March 2023 and June 2024. Abdominal aortic calcification maps were generated by subtracting VNCa from the corresponding CTA images to calculate the abdominal calcification volume (ACV) and aortic wall volume (AWV). Percentage calcification volume (PCV) was calculated as ACV/AWV. Agatston scores from nCE-CCT classified patients into low- (≤ 100) and high-risk (> 100) CVD groups. Correlations between Agatston score, ACV, and PCV were analyzed using Spearman’s rank correlation, and receiver operating characteristic analysis was used to determine the performance and cutoff values of ACV and PCV, with McNemar’s test comparing sensitivities and specificities.

**Results:**

The study included 200 patients, 163 low- and 37 high-risk patients. Agatston score correlations with ACV and PCV were 0.75 and 0.78, respectively (*p* < 0.0001). PCV showed a superior AUC (0.94) than ACV (0.90, *p* = 0.0002). Cutoff values were 5.74 mL for ACV (75.7% sensitivity, 89.0% specificity) and 14.81% for PCV (73.0% sensitivity, 99.4% specificity), and PCV specificity was significantly higher than ACV specificity (*p* < 0.0001).

**Conclusion:**

PCV > 14.81% indicates an increased CVD risk, suggesting that PCV is a potential imaging biomarker for high-risk patients with CVD. Abdominal CTA alone may identify high-risk patients with CVD, warranting further cardiovascular screening.

**Supplementary Information:**

The online version contains supplementary material available at 10.1007/s11547-025-01978-0.

## Introduction

The increasing prevalence of atherosclerotic diseases, especially coronary artery disease, is a significant public health concern [1, 2]. The Agatston score quantifies coronary artery calcification, predicting future cardiovascular disease (CVD) events such as coronary heart disease, cerebrovascular disease, peripheral vascular disease, and heart failure [3]. While the assessment of coronary calcification is widely accepted, the evaluation of abdominal atherosclerosis severity lacks a consensus. Abdominal atherosclerosis rarely causes intestinal ischemia from superior mesenteric artery stenosis and secondary hypertension due to renal artery stenosis, but these are less prevalent and uncommon than coronary artery disease [4, 5]. This view may complicate the assessment of abdominal atherosclerosis severity.

The increased frequency of CT scans has led to more incidental detection of abdominal atherosclerosis using abdominal CT scans [6]. Accurately assessing abdominal aortic calcification via abdominal CT scans and predicting the severity of coronary artery calcification remains a medical challenge, as chest CT is often avoided because of radiation concerns. The clinical issue that needs to be clarified is the severity of abdominal atherosclerosis in relation to the risk of CVD.

Several studies have examined the relationship between abdominal aortic and coronary arterial calcification, indicating that abdominal artery calcification is correlated with a higher risk of CVD [7–9]. However, a comprehensive method for calculating abdominal aortic calcification has yet to be developed. A previous study used CT angiography (CTA) to grade the severity of aortic calcification in the distal abdominal aorta [10]; however, these methods are semiquantitative and imprecise. This grading process relies on subjective visual assessment by radiologists, which can lead to interobserver variability. Furthermore, this approach does not consider the three-dimensional spatial distribution of calcifications, which may be clinically important. Given these limitations, more objective and quantitative methods are needed to improve the accuracy and reproducibility of abdominal aortic calcification assessment.

Photon-counting CT (PCCT) has recently been introduced into clinical practice and is increasingly being used for abdominal imaging. Unlike conventional energy-integrating detectors, PCCT detectors convert X-ray photons directly into electrical signals without the use of scintillators. PCCT detectors do not require a septum and have smaller detector pixels than conventional detectors; therefore, PCCT offers improved spatial resolution. In addition, PCCT eliminates the energy weighting effect, resulting in improved dose efficiency and superior spectral resolution [11]. Recent studies have shown that PCCT effectively removes calcium in clinical settings [12, 13]. One study demonstrated that a virtual non-calcium (VNCa) algorithm with PCCT successfully removed highly calcified plaques, enhancing the coronary CTA image clarity [12]. Another study highlighted the efficacy of PCCT in reducing calcification blooming artifacts and improving the spatial resolution [13]. The virtual non-iodine (VNI) algorithm is another method for quantifying calcification on CTA. In the cardiovascular field, there were reports of the use of the VNI algorithm based on PCCT to quantify calcification [14, 15]. However, research on the application of these algorithms to the abdominal region for clinical purposes is limited. Moreover, accurate assessment of abdominal aortic calcification requires the measurement of the aortic calcification volume (ACV) and its proportion in the abdominal aortic wall volume (AWV). The percentage calcification volume (PCV) is obtained by calculating the ratio of ACV to AWV. These parameters can be assessed by applying the VNCa algorithm based on PCCT angiography. To date, there have been no reports on the evaluation of the degree of aortic calcification by calculating the ratio of ACV to AWV, such as PCV. ACV is an absolute indicator of the volume of calcification in the abdominal aorta. However, AWV varies from patient to patient depending on body size, and PCV can provide a standardized value for the calcification indicator. PCV may provide a new and potentially valuable perspective for accurately assessing the degree of abdominal aortic calcification. Accurate assessment of abdominal aortic calcification may help identify individuals at higher risk of CVD, allowing for more targeted preventive interventions and improved patient management.

The aim of this study was to measure abdominal aortic calcification by volumetric analysis using a PCCT angiography-based VNCa and VNI algorithms and to investigate the correlation between ACV and PCV and the Agatston score. The final goal was to use ACV and PCV to differentiate between grades and low- and high-risk groups in terms of CVD risk based on the Agatston score and to clarify the imaging biomarker of abdominal atherosclerosis in high-risk patients with CVD.

## Materials and methods

### Participants enrollment

This retrospective study was approved by the Institutional Review Board of our university hospital. The requirement for informed consent was waived because this study was retrospective and non-interventional. Patients who underwent upper abdominal multiphasic contrast-enhanced CT, including CTA with a PCCT scanner, between March 2023 and June 2024 were included. The patient group between March 2023 and April 2023 included a patient cohort that overlaps with a previous study (n = 12); however, this study is a unique study on the theme of abdominal atherosclerosis, and the theme is completely different from past study [16]. A previous study examined how low-keV virtual monoenergetic imaging (VMI) could be used to visualize the periphery of abdominal arterial branches; however, this study is a completely different topic and provides truly novel data by accurately quantifying calcification of the abdominal aorta.

### Exclusion criteria

Patients who did not undergo non-contrast-enhanced chest CT (nCE-CCT) within 180 days of the abdominal CTA scan, those who underwent abdominal aortic surgery, and those without available vascular spectral post-processed (VSPP) image data of abdominal CTA.

**Fig. 1:**
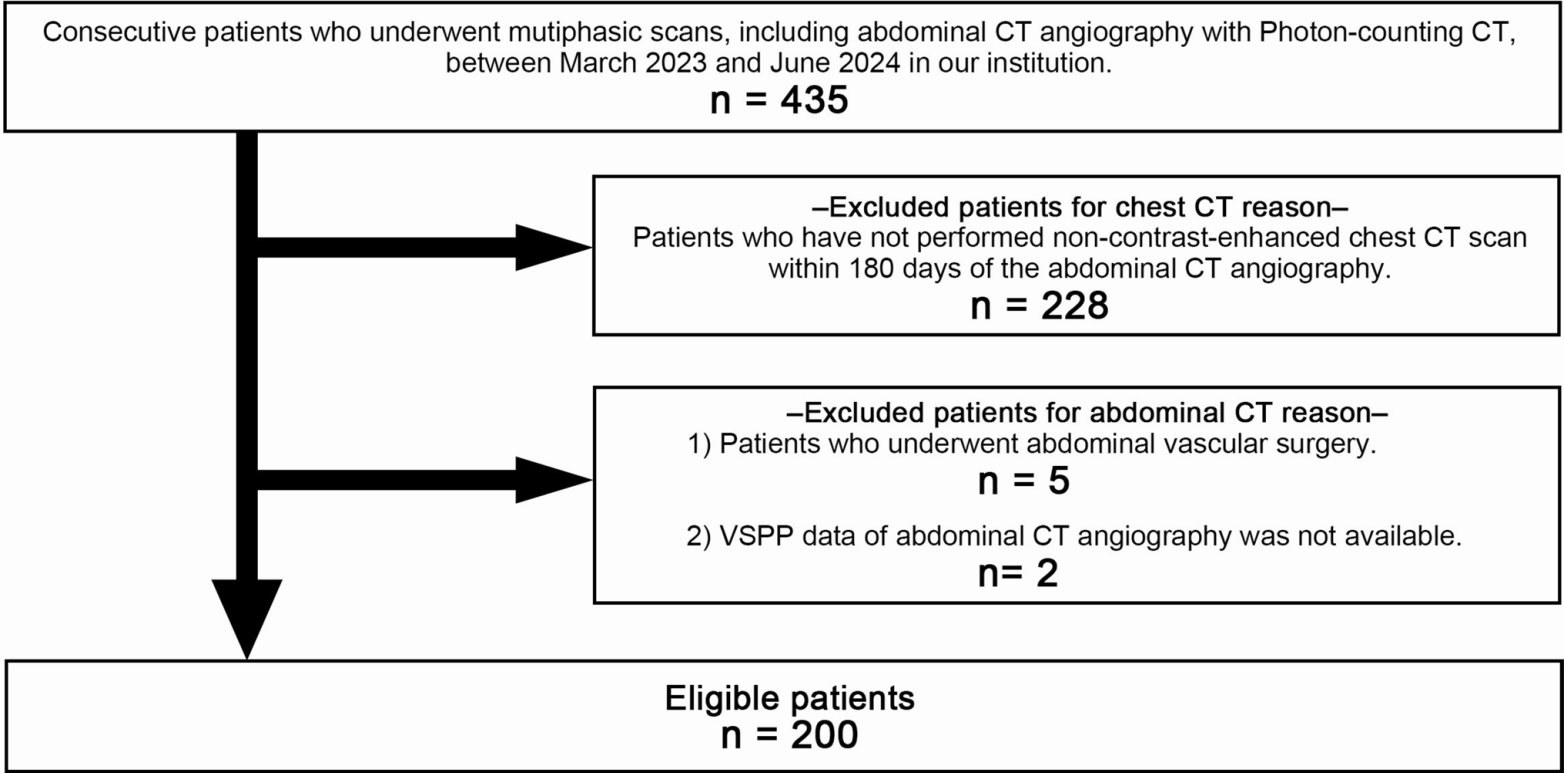
Patient flowchart

**Fig. 2:**
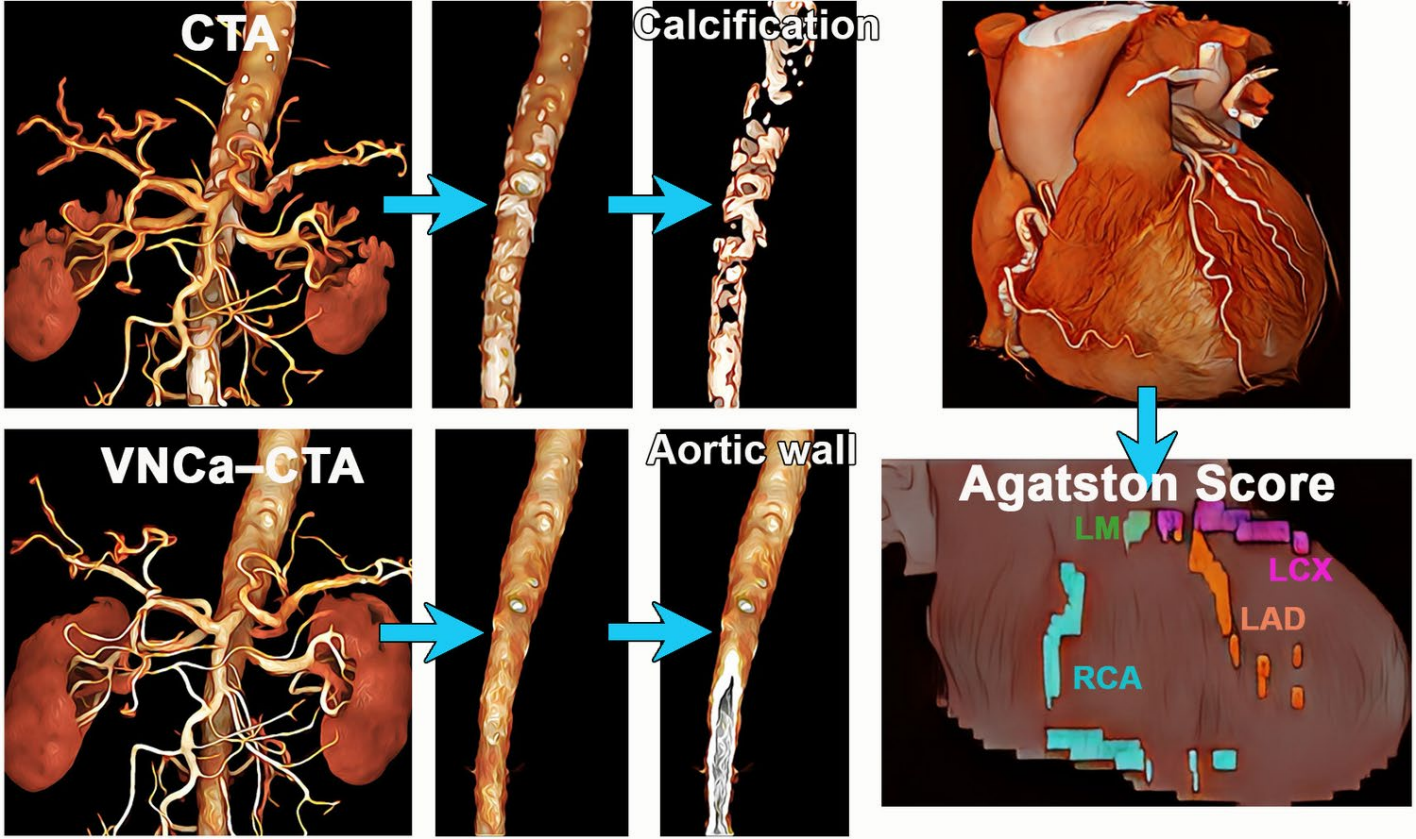
Research design. Calcification in the abdominal aortic wall was extracted from 3D CTA images and aortic calcification volume (ACV) was measured. In addition, the aortic wall was constructed from 3D CTA images with VNCa applied and aortic wall volume (AWV) was measured. Percentage calcification volume (PCV) is the ratio of ACV to AWV. Coronary artery calcification was quantified using a dedicated workstation to calculate the Agatston score. Using the Agatston score indicators as outcomes, we investigated the relationship between ACV and PCV and attempted to define the severity of abdominal atherosclerosis. CTA = CT angiography; VNCa = virtual non-calcium

The summarized information is presented in Fig. 1 and Table 1. Figure 2 illustrates the research design of this study.

**Table 1:** Characteristics of eligible patients

Sample size	200
Age	67.7 ± 14.0 (range, 19–87) years
Sex	Male: 115, Female: 85
CT scanner of non-contrast-enhanced chest CT	
NAEOTOM Alpha (Siemens)	139 (69.5%)
Aquilion Precision (Canon)	22 (11.0%)
Revolution CT (GE Healthcare)	15 (7.5%)
Aquilion One (Canon)	24 (12.0%)
Intervals between abdominal CTA and chest CT	21.5 ± 40.6 (range, 0–179) days
Agatston score grade	
Grade 0 (Agatston Score: 0)	102 (51.0%)
Grade 1 (Agatston Score: 1–99)	61 (30.5%)
Grade 2 (Agatston Score: 100–299)	12 (6.0%)
Grade 3 (Agatston Score: > 300)	25 (12.5%)
Risk of cardiovascular disease events	
Low risk (Agatston Score ≤ 100)	163 (81.5%)
High risk (Agatston Score > 100)	37 (18.5%)

### CT scan protocol

#### Abdominal CT angiography

A clinical dual-source PCCT scanner (NAEOTOM Alpha; Siemens Healthineers, Forchheim, Germany) was operated at 120 kV with 120 × 0.2 mm collimation, a 0.8 pitch factor, and a 0.50 s gantry rotation time [16]. Combined Applications to Reduce Exposure (CARE) Dose 4D (Siemens Healthineers) automatically modulated the tube current, targeting an image quality of 170. In the upper abdominal multiphasic CT, 600 mgI/kg of nonionic iodine contrast (Iomeron 350; Eisai, Tokyo, Japan) was injected for 25 s. Bolus tracking in the abdominal aorta began early arterial phase scanning after an 8 s delay at a 40 HU threshold. CTA images were reconstructed axially using QIR (Quantum Iterative Reconstruction; Siemens Healthineers) at level 4 (maximum level), Qr44 (quantitative regular) kernel, 345 mm field of view, and 512 × 512 matrix. VSPP images (1.0 mm thickness, no gaps) were transferred to a picture archiving and communication system. VSPP images can be used to create VMI at any energy level (40–140 keV) or to create material decomposition images. A radiologist A (12-year experience in abdominal imaging and 3D image processing) created VNCa images, called PureLumen (Siemens Healthineers), and VNI images from abdominal CTA VSPP images (70 keV) using a CT scanner console (syngo. CT VA50; Siemens Healthineers). The 70 keV energy level was chosen because the contrast was comparable to that of conventional 120 kVp CT [17].

#### Non-contrast-enhanced chest CT

nCE-CCT images were acquired with four CT systems: NAEOTOM Alpha, Aquilion Precision (Canon Medical Systems, Otawara, Japan), Revolution CT (GE Healthcare, Waukesha, WI, USA), and Aquilion One (Canon Medical Systems). The parameters for each scanner are listed in Table 2.

**Table 2:** Scan protocols for non-contrast-enhanced chest CT

CT scanner	Tube voltage/current	Rotation time/pitch	Thickness/gap	Kernel	Iterative reconstruction
NAEOTOM Alpha (Siemens)	120 kV/AEC (IQ 150)	0.25 s/0.88	3.0 mm/0.0 mm	Qr40	QIR (level 4)
Aquilion Precision (Canon)	120 kV/AEC (SD 12)	0.50 s/0.806	3.0 mm/0.0 mm	F04-H	AIDR3D eMILD
Revolution CT (GE Healthcare)	120 kV/AEC (NI 11.5)	0.50 s/0.992	3.0 mm/0.0 mm	HD standard	ASiR-V 30%
Aquilion One (Canon)	120 kV/AEC (SD 12)	0.50 s/0.813	3.0 mm/0.0 mm	FC04	AIDR3D MILD

AEC = Automatic exposure control; IQ = Image quality; SD = Standard deviation; NI = Noise index; QIR = Quantum iterative reconstruction; AIDR = Adaptive iterative dose reduction; ASiR = Advanced statistical iterative reconstruction

### Abdominal aortic calcification evaluation

**Fig. 3:**
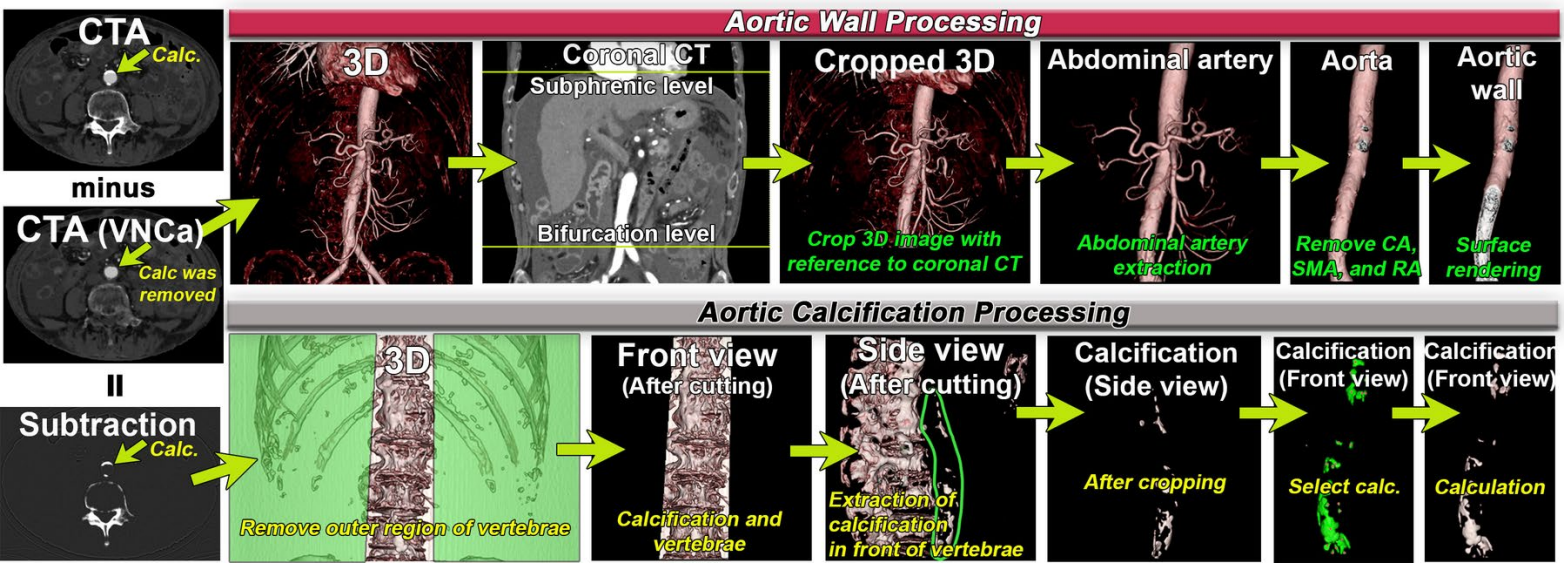
How to process 3D Images. A schematic diagram of the procedure for generating abdominal aortic calcifications and the abdominal aortic wall from 3D CTA images. The top row explains the procedure for creating an abdominal aortic wall from VNCa CTA images. The lower part of the figure shows the procedure for extracting abdominal aortic calcification. Using a subtraction image created by subtracting the VNCa image from the corresponding CTA image, we extracted the calcification of the abdominal aortic wall according to the procedure shown in the Figure. CTA = CT angiography, VNCa = virtual non-calcium

Quantification of ACV and its proportion in the AWV was performed by the radiologist A and another one radiologist B (12-year experience in abdominal imaging) using a 3D image analysis system (SYNAPSE VINCENT version 6.7; Fujifilm, Tokyo, Japan) on the abdominal 3D CTA images using the VNCa algorithm. Radiologist A also quantified ACV on 3D CTA images using the VNI algorithm. Figure 3 outlines the methodology for extracting abdominal aortic calcification and abdominal aortic wall from 3D images.

#### Abdominal aortic calcification processing (VNCa and VNI)

1. VNCa and VNI images were generated from CTA images using the VNCa and VNI algorithms.
2. VNCa images were subtracted from the corresponding abdominal CTA images to create subtraction images. The term “subtraction images” refers to images that show only the calcification and the remaining parts of the bone.
3. Abdominal aortic calcification 3D images were generated from the VNCa subtraction images and VNI images using a workstation. At this stage, calcification and the remaining bone were displayed as 3D images.
4. Coronal CTA images with edges at the diaphragm and aortic bifurcation were used as references. Referring to these coronal CTA images, 3D images at the level cranial to the diaphragm and caudal to the aortic bifurcation were removed.
5. Regions without vertebrae on both sides of the vertebral body were removed from the frontal view. By performing this procedure, only the calcification of the abdominal aorta and vertebrae remained on the 3D image.
6. The 3D image was rotated to view and extract the abdominal aortic calcification using a selection tool. By clicking on the calcification with the selection tool on the workstation, we extracted each calcification. Through these processes, the calcification of the abdominal aorta was extracted as a 3D image.
7. Finally, abdominal aortic calcification 3D images were analyzed and ACV [mL] values were measured.

#### Abdominal aortic wall processing (VNCa only)

1. 3D abdominal artery images without calcification were generated from VNCa CTA images by setting boundaries at the diaphragm and aortic bifurcation in the coronal section. It is impossible to obtain a 3D image of a blood vessel using the VNI algorithm. Therefore, we decided to construct an aortic wall using only the VNCa algorithm.
2. The celiac, superior mesenteric, renal, and major lumbar arteries were manually removed to create the abdominal aorta.
3. The aortic lumen was removed by surface rendering the 3D image and performing thick surface extraction at a scale of 1.5 mm to create the abdominal aortic wall. The thickness of 1.5 mm was chosen because previous reports have shown that the thickness of the abdominal aortic wall ranges from 1.48 to 1.78 mm [18, 19].
4. The volume of the abdominal aortic wall was measured to obtain AWV [mL]. The percentage of ACV to AWV was calculated as PCV [%] using the following formula:
  $$\text{PCV} \left(\%\right)=\frac{\text{ACV }\left(\text{mL}\right)}{\text{AWV }\left(\text{mL}\right)} \times 100$$

### Coronary artery calcification scoring

The Agatston score was determined using an nCE-CCT scan with a 3.0 mm slice thickness to assess coronary artery calcification levels [20]. SYNAPSE VINCENT calcification analysis of the cardiac region automatically calculated scores. The radiologist A, who was blinded to the ACV and PCV parameters, conducted all Agatston score measurements. Agatston score was classified as follows [21]:

- Grade 0 (Agatston score 0): very low risk, no detectable coronary artery calcification.
- Grade 1 (Agatston score 1–99): mild risk, mild coronary artery calcification.
- Grade 2 (Agatston score 100–299): moderate risk, moderate coronary artery calcification.
- Grade 3 (Agatston score > 300): severe risk, extensive coronary artery calcification.

### Relationship between aortic calcification volume, percentage calcification volume, and Agatston score

Correlation coefficients were calculated for ACV and PCV derived from the VNCa algorithm, and ACV derived from the VNI algorithm with Agatston score.

In addition, multiple comparisons were made for the values of ACV and PCV for each Agatston score grade (grades 0 to 3). The measurements taken by the two radiologists were used to calculate interobserver reliability, and the average of the two values was used for multiple comparisons.

### Differentiation for the risk of cardiovascular disease events

Previous studies have shown that an Agatston score > 100 is associated with a higher risk of CVD [22–24]. We categorized AS ≤ 100 as low risk and AS > 100 as high risk for CVD using ACV and PCV.

Furthermore, to investigate the impact of the Agatston score due to differences in CT scanners, we performed subgroup analysis to determine the ability of each of the four CT scanners to differentiate between CVD risk in ACV and PCV.

### Statistical analysis

Because the Agatston score, ACV, and PCV showed non-normal distributions in the Shapiro–Wilk test, all statistical analyses were performed using nonparametric tests. Spearman’s rank correlation coefficients (ρ) were calculated for ACV and PCV and Agatston score [25]. The ρ values of the VNCa and VNI algorithms were compared using Williams’ t tests. The measurements taken by the two radiologists were evaluated for interobserver reliability by calculating the intraclass correlation coefficients (ICC) [26]. The Kruskal–Wallis test with post hoc Steel–Dwass tests was used to compare the ACV and PCV values across Agatston score grades. The ability to differentiate between each grade was calculated using receiver operating characteristic (ROC) analysis and the area under the curve (AUC). The AUCs of ACV and PCV for differentiating each grade were compared using the DeLong test. In addition, to differentiate between the low- and high-risk groups of CVD, the AUCs of ACV and PCV for differentiating the two groups were calculated, and the two were compared using the DeLong test. In addition, we used the Youden index to calculate the appropriate cutoff values for ACV and PCV and calculated the sensitivity, specificity, and accuracy of differentiating the two groups based on the cutoff values. The sensitivity, specificity, and accuracy of ACV and PCV were compared using the McNemar test. Williams’ t test was performed using the R software (version 4.4.2, The R Foundation for Statistical Computing). All other statistics were performed using JMP Pro 12.2 (SAS Institute Inc., Cary, USA). A *p* value of less than 0.05 was considered statistically significant.

## Results

### Patient population

Of the 435 patients, 235 met the exclusion criteria (228 for chest CT reason and 7 for abdominal CT reasons), including 200 eligible patients (Fig. 1). Table 1 summarizes the details, such as age, sex proportion, interval between abdominal CTA and nCE-CCT, Agatston score grade ratio, and low- to high-risk CVD group ratios. The specific CT scanners used for nCE-CCT are listed in Table 1.

### Correlation between abdominal aortic calcification and Agatston score

**Fig. 4:**
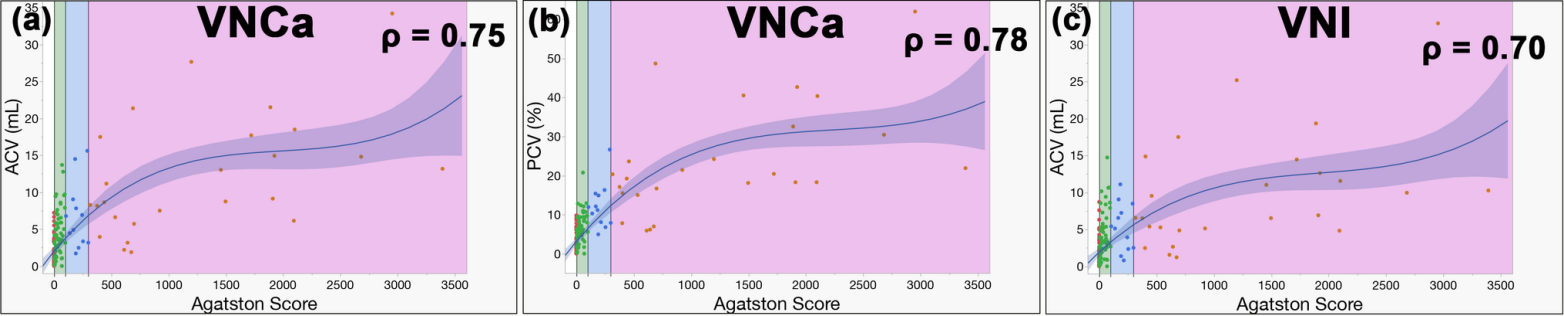
Correlations between ACV, PCV, and Agatston scores. Scatter plots showing the correlation between ACV, PCV, and the Agatston score. Data points are color-coded by grade: red for grade 0, green for grade 1, blue for grade 2, and orange for grade 3. The regression curve represents the relationship between the parameters, with the shaded blue area depicting the 95% confidence interval around the curve. **a** The x-axis indicates the Agatston score, and the y-axis shows ACV (mL). The correlation coefficient (ρ) between ACV and the Agatston score for the VNCa algorithm is 0.75, indicating a strong positive correlation. **b** The x-axis indicates the Agatston score, and the y-axis shows PCV (%). The correlation coefficient (ρ) between PCV and the Agatston score for the VNCa algorithm is 0.78, also indicating a strong positive correlation. **c** The x-axis indicates the Agatston score, and the y-axis shows ACV (mL). The correlation coefficient (ρ) between ACV and the Agatston score for the VNI algorithm is 0.70, indicating a strong positive correlation. ACV = Aortic calcification volume [mL]; PCV = percentage calcification volume [%]; VNCa = virtual non-calcium; VNI = virtual non-iodine

ρ showed strong correlations between the Agatston score and ACV (ρ = 0.75, 95% confidence interval [C.I.]: 0.67–0.80, *p* < 0.0001) and PCV (ρ = 0.78, 95% C.I.: 0.71–0.84, *p* < 0.0001) for the VNCa algorithm. ρ showed strong correlation between the Agatston score and ACV (ρ = 0.70, 95% C.I.: 0.63–0.77, *p* < 0.0001) for the VNI algorithm. There was no statistically significant difference in the ρ of ACV using VNCa compared to the ρ of ACV using the VNI algorithm (*p* = 0.37). There was also no statistically significant difference in the ρ of PCV using VNCa compared to the ρ of ACV using the VNI algorithm (*p* = 0.071). Figure 4 shows scatter plots of the correlation between the Agatston score and ACV, and between the Agatston score and PCV for the VNCa and VNI algorithms.

### Parameter differences of abdominal aortic calcification in each Agatston score grade

The ICC for the ACV measured by the two radiologists was 1.00 (95% C.I., 1.00–1.00), and the ICC for the PCV was 0.93 (95% C.I., 0.86–0.96). Both the values showed excellent reliability. The average values of ACV and PCV measured by the two radiologists were calculated and used in subsequent analyses.

The median (interquartile range, IQR) values of grades 0 to 3 of the Agatston score for ACV were 0.24 (0, 1.44), 3.57 (1.42, 5.86), 6.61 (3.26, 8.71), and 9.09 (6.22, 17.84), respectively. For PCV, the values were 0.51 (0, 2.81), 7.46 (3.26, 10.67), 13.11 (8.05, 17.39), and 21.69 (16.76, 36.77), respectively.

When multiple comparisons were made for each grade of Agatston score, a statistically significant difference was observed for both ACV and PCV (both *p* < 0.0001). When comparing each grade, for ACV, significant differences were observed between all groups except for grades 1 and 2 (*p* = 0.17) and grades 2 and 3 (*p* = 0.16) (except these, all *p* < 0.0001). For PCV, there were significant differences between all groups (*p* = 0.0094 between grades 1 and 2, *p* = 0.026 between grades 2 and 3, and all *p* < 0.0001 for the others).

The AUC values for differentiating each grade from the other grades were 0.89, 0.76, 0.78, and 0.91 for ACV in the order of grades 0 to 3. For PCV, the AUCs were 0.90, 0.80, 0.84, and 0.95. When the AUCs of ACV and PCV were compared, the AUCs of grade 2 and grade 3 were significantly higher for PCV than for ACV (*p* = 0.014, 0.0009).

**Fig. 5:**
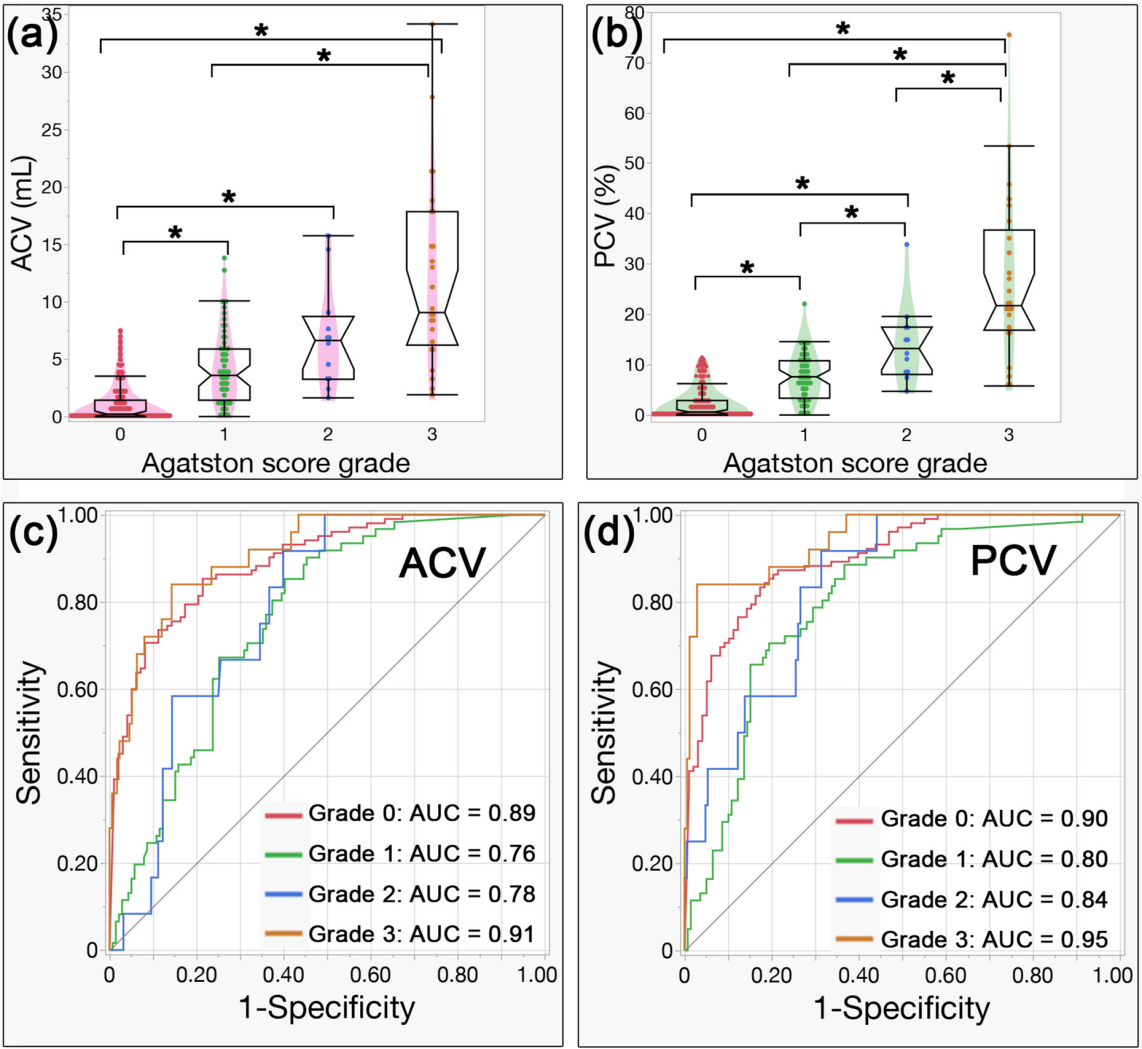
Box plots and ROC curves of ACV and PCV across Agatston score grades. Box plots of ACV and PCV across Agatston score grades (0–3) and receiver operating characteristic (ROC) curves for distinguishing each grade from others. * An asterisk indicates a statistically significant difference. **a** ACV values increase with higher Agatston score grades, showing statistically significant differences between all grades except grades 1 vs. 2 and grades 2 vs. 3. **b** PCV values also increase with higher grades, with statistically significant differences observed between all grades. **c**, **d** ROC curves show the performance of ACV and PCV in discriminating each grade from the others, with AUC values listed at the bottom right of each plot. ACV = Aortic calcification volume; PCV = percentage calcification volume; AUC = area under the curve

The median values (IQR), multiple comparisons, and AUCs of each Agatston score grade for ACV and PCV are detailed in Table 3 and are illustrated in the box plots and ROC curves in Fig. 5.

**Table 3:** Multiple comparisons of ACV and PCV in each Agatston score grade

	ACV (mL)	PCV (%)	*p*-value
Grade 0	0.24 (0–1.44)	0.51 (0–2.81)	
Grade 1	3.57 (1.42–5.86)	7.46 (3.26–10.67)	
Grade 2	6.61 (3.26–8.71)	13.11 (8.05–17.39)	
Grade 3	9.09 (6.22–17.84)	21.69 (16.76–36.77)	
*p*-value	< 0.0001*	< 0.0001*	
Grade 0 vs 1	< 0.0001*	< 0.0001*	
Grade 0 vs 2	< 0.0001*	< 0.0001*	
Grade 0 vs 3	< 0.0001*	< 0.0001*	
Grade 1 vs 2	0.17	0.0094*	
Grade 1 vs 3	< 0.0001*	< 0.0001*	
Grade 2 vs 3	0.16	0.026*	
AUC (95% C.I.)			
Grade 0 vs 1, 2, 3	0.89 (0.84, 0.93)	0.90 (0.85, 0.94)	0.12
Grade 1 vs 0, 2, 3	0.76 (0.69, 0.82)	0.80 (0.73, 0.86)	0.091
Grade 2 vs 0, 1, 3	0.78 (0.67, 0.86)	0.84 (0.73, 0.91)	0.014*
Grade 3 vs 0, 1, 2	0.91 (0.84, 0.95)	0.95 (0.88, 0.98)	0.0009*

ACV = Aortic calcification volume; PCV = percentage calcification volume; AUC = area under the curve: 95% C.I. = 95% confidence interval

^*^The asterisks indicate statistically significant differences

### Differentiation of the risk for cardiovascular disease events

For ACV, the median values (IQR) for the low- and high-risk groups for CVD were 1.00 (0.093, 3.55) and 8.43 (5.14, 14.82), respectively. The median values (IQR) for PCV were 2.25 (0.18, 7.56) and 19.86 (11.23, 30.17), respectively.

The AUCs for differentiating between the low- and high-risk groups for CVD of ACV and PCV were 0.90 and 0.94, respectively. The AUC for PCV was significantly higher than that for ACV (*p* = 0.0002). The optimal cutoff values for differentiating between the two groups were 5.74 mL for ACV and 14.81% for PCV. Using these cutoff values, ACV was able to differentiate between the two groups with a sensitivity of 75.7%, specificity of 89.0%, and accuracy of 86.5%. In contrast, PCV was able to differentiate between the two groups with a sensitivity of 73.0%, specificity of 99.4%, and accuracy of 94.5%. The specificity and accuracy of PCV were significantly higher than those of ACV (both *p* < 0.0001).

**Fig. 6:**
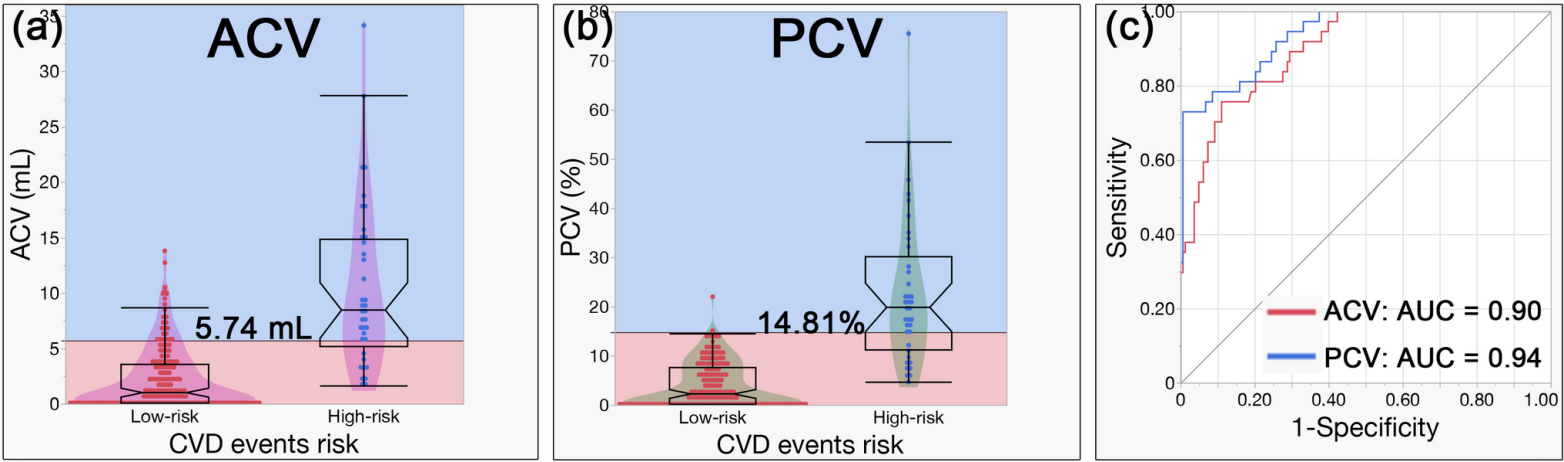
Box plots and ROC curves of ACV and PCV for differentiating low risk and high risk for CVD events. Box plots of ACV and PCV stratified into low- and high-risk groups for CVD events, along with receiver operating characteristic (ROC) curves for distinguishing between these groups. **a**, **b** Box plots display ACV and PCV values, with red dots representing the low-risk group and blue dots the high-risk group. Cutoff values are indicated: ACV = 5.74 mL and PCV = 14.81%. The red-shaded area shows values below the cutoff, while the blue-shaded area shows value above it. PCV has higher specificity, as shown by more red points in the red area compared to ACV. **c** ROC curves illustrate the performance of ACV and PCV in differentiating between risk groups, with AUCs of 0.90 for ACV and 0.94 for PCV. ACV = Aortic calcification volume; PCV = percentage calcification volume; CVD = cardiovascular disease; AUC = area under the curve

Table 4 summarizes the above results. Figure 6 shows box plots and ROC curves for ACV and PCV in differentiating the risk for CVD.

**Table 4:** Diagnostic performance comparison between ACV and PCV for differentiating CVD events risk

	ACV (mL)	PCV (%)	*p*-value
Low risk	1.00 (0.093, 3.55)	2.25 (0.18, 7.56)	
High risk	8.43 (5.14, 14.82)	19.86 (11.23, 30.17)	
AUC (95% C.I.)	0.90 (0.84, 0.94)	0.94 (0.89, 0.97)	0.0002*
Cutoff value	5.74 mL	14.81%	
Sensitivity	75.7% (28/37)	73.0% (27/37)	0.32
Specificity	89.0% (145/163)	99.4% (162/163)	< 0.0001*
Accuracy	86.5% (173/200)	94.5% (189/200)	< 0.0001*

ACV = Aortic calcification volume; PCV = percentage calcification volume; AUC = area under the curve: 95% C.I. = 95% confidence interval

^*^The asterisks indicate statistically significant differences

### Subgroup analysis to differentiate the risk of CVD events according to differences in CT scanners

In NAEOTOM Alpha, the AUC for ACV was 0.89, and the AUC for PCV was 0.94, with PCV being significantly higher (*p* = 0.0002). The accuracy of differentiating CVD risk was 86.3% and 95.0% for ACV and PCV, respectively. PCV was significantly higher than ACV (*p* = 0.0005).

At Aquilion Precision, the AUC for ACV was 0.93, and the AUC for PCV was 0.95, with PCV being higher, but there was no statistically significant difference (*p* = 0.75). The specificities of ACV and PCV were 66.7% and 93.3%, respectively, and that of PCV was significantly higher (*p* = 0.046). The accuracies of ACV and PCV were 77.3% and 90.9%, respectively, and PCV was significantly higher (*p* = 0.025).

For Revolution CT and Aquilion ONE, there were no statistically significant differences in AUC, sensitivity, specificity, and accuracy between ACV and PCV, but all parameters showed similar or higher values for PCV than for ACV.

The details of these results are presented in the Supplementary Table.

## Discussion

In our study, we applied the PCCT-based VNCa and VNI algorithms to quantify abdominal aortic calcification and to clarify the severity of abdominal arterial atherosclerosis using the risk of CVD as an outcome. We calculated ACV and PCV as indicators of abdominal atherosclerosis, which showed strong correlations with Agatston score. When comparing Agatston score grades, PCV showed significant differences between all grades, and with increase in grade, PCV showed a significantly higher value. In addition, the AUC for discriminating grades 3 and 4 from other grades was significantly higher for PCV than for ACV (ACV: 0.78 and 0.91, PCV: 0.84 and 0.95). In addition, the AUC for discriminating between low- and high-risk groups for CVD was significantly higher for PCV (0.94) than for ACV (0.90). The optimal cutoff values to discriminate between the two groups were 5.74 mL for ACV and 14.81% for PCV. Using these cutoff values, ACV could discriminate between the two groups with a sensitivity of 75.7%, a specificity of 89.0%, and an accuracy of 86.5%, and PCV could discriminate between the two groups with a sensitivity of 73.0%, a specificity of 99.4%, and an accuracy of 94.5%. The specificity and accuracy of PCV were significantly higher than those of ACV. No reports have calculated the threshold values of abdominal aortic calcification parameters and examined the severity of abdominal atherosclerosis.

In our study, the ability to discriminate the risk of CVD events was significantly higher for PCV than for ACV. The first explanation for this is the relative assessment advantage of PCV. For example, even with the same ACV, the degree of atherosclerosis may differ between patients with large and small aortas, but PCV is thought to be able to correct for this effect and provide a more accurate assessment of atherosclerosis. In addition, PCV more accurately reflects the extent of calcification. As calcification spreads across the aortic wall, the flexibility of the aorta decreases and the ability to maintain smooth blood flow is lost, increasing the risk of developing CVD events [27]. PCV may be a more direct indicator of structural and functional abnormalities in the abdominal aorta and the severity of abdominal atherosclerosis. In addition, the specificity of PCV for differentiating the risk of CVD events was significantly higher than that of ACV. This indicates that PCV has a high ability to accurately identify low-risk group, suggesting that PCV is an indicator that is not dependent on differences in abdominal aortic size among patients. Because ACV is purely a volume, it may overestimate the degree of atherosclerosis in patients with large aortic volumes. As a result, the specificity of ACV was thought to be significantly inferior to PCV.

Reddy et al. used abdominal CTA to measure the extent of abdominal aortic calcification in a cohort of 75 patients [10]. Three radiologists assessed the severity of abdominal aortic calcification in the distal abdominal aorta, specifically from the inferior mesenteric artery to the level of the bifurcation, using a six-point grading system. A vascular-specific workstation was used to detect calcification using a threshold of CT values (450 HU) to calculate the abdominal aortic calcification score, which correlated strongly with radiologist scores. This measurement is based on subjective scoring by radiologists and is considered a semiquantitative and imprecise method of measurement. In contrast, we used the PCCT-based VNCa algorithm to measure the volume of calcification in the abdominal aorta and determined the ratio of the volume of calcification to the volume of the aortic wall to assess the degree of calcification. We believe that our measurement method is more detailed and accurate for assessing abdominal atherosclerosis.

We measured ACV using the VNCa and VNI algorithms, and there was no significant difference in the correlation with the Agatston score for either algorithm; therefore, it seems that there is no significant impact on the clinical setting of the results of ACV as the outcome Agatston score. The advantage of the VNCa algorithm is that it can calculate not only ACV but also PCV because it can calculate AWV because it can obtain images of blood vessels that have had calcification removed. One of its disadvantages is that because it is an image that removes calcification, it cannot be used as an alternative to non-contrast-enhanced CT. In contrast, the VNI algorithm has the advantage of potentially replacing non-contrast-enhanced CT because it removes iodine. Previous studies have reported that VNI closely matches the calcification score on non-contrast CT [14, 15]. The disadvantage of the VNI algorithm is that it cannot calculate PCV because it cannot calculate AWV.

We did not evaluate the accuracy of the PCCT angiography-based VNCa and VNI algorithms for detecting calcification by comparing calcification extraction with the CT value threshold on non-contrast-enhanced CT. Some studies have set the threshold for calcification detection at 130 HU on non-contrast-enhanced CT images [28–30]. However, another study reported that the appropriate threshold was 299 HU [31]. Because calcification quantification on non-contrast-enhanced CT images is not always an accurate indicator due to threshold variability, it is difficult to confirm the accuracy of VNCa and VNI algorithms. PCCT uses spectral information to separate materials based on their specific energy attenuation profiles. This allows calcium to be distinguished from other materials such as iodine and soft tissue [15, 32]. VNCa algorithm reconstructs images by synthesizing monoenergetic and non-calcium images. It computes a calcium map that helps to virtually remove calcium while preserving other materials, especially iodine [32]. This algorithm can produce an image in which calcium is effectively removed, allowing clearer visualization of vascular structures without calcification artifacts [32]. The advantage of the VNCa algorithm is that it can reduce blooming artifacts from calcified plaques, potentially improving the accuracy of stenosis quantification in coronary CTA [12, 32]. However, there may be issues with underestimation of calcifications compared to non-contrast-enhanced images, and further optimization is needed to ensure reliable clinical applications [15]. The aim of this study was to quantify abdominal aortic calcification using only abdominal CTA, and we believe that the VNCa algorithm, which can effectively and easily remove calcium from CTA images, has a high clinical utility.

In our study cohort, there were 163 low-risk cases and 37 high-risk cases of CVD, indicating an imbalanced data set. Equal sample sizes are typically preferred because they increase statistical power, thereby increasing the likelihood of correctly rejecting a false null hypothesis while decreasing the risk of type I error (incorrectly rejecting a true null hypothesis) [33]. In contrast, different sample sizes can lead to biased estimates and reduced power, especially if the variances between groups differ significantly [33]. Although equal sample sizes are usually optimal to increase statistical power and simplify analysis, it is sometimes more appropriate to maintain a natural balance that accurately represents the different incidence rates between groups [34]. In a large epidemiologic study called the Framingham Heart Study, 3969 men and 4522 women aged 30 to 74 years were examined over a 12-year period and reported that CVD events occurred in 718 men (18.1%) and 456 women (10.1%) [35]. In this study, the high-risk group for CVD events accounted for 18.5% of the total, which was broadly consistent with the epidemiologic study. Therefore, we assumed that the difference in sample size between the two groups was due to the difference in morbidity rates. Moreover, there are methods for correcting imbalanced datasets, such as oversampling the minority group; however, if they are not implemented carefully, there is a risk of overfitting and other miscalibrations of the prediction probability [36]. While correcting imbalances is important, maintaining the characteristics of real-world data is also important for ensuring clinical applicability. If possible, the challenge for the future is to verify the results of this study using a larger dataset.

This study had several limitations. First, it was performed at a single institution with a relatively small sample size. Second, the optimal imaging conditions for Agatston score measurement were nCE-CCT images with ECG-gated axial thickness of 3.0 mm [21]. In our study, nCE-CCT images were reconstructed to 3.0 mm, but ECG synchronization was not performed. A previous study reported the effect of increased heart rate on the measurement of Agatston score in non-ECG-gated CT [37]. In non-ECG-gated CT scans, the Agatston score is often underestimated compared to ECG-gated CT scans [38]. In addition, because non-ECG-gated CT scans are not synchronized with the cardiac cycle, they are more likely to produce motion artifacts, which may reduce the accuracy and reliability of coronary artery calcification detection and quantification [39]. However, recent advances in non-ECG-gated CT techniques, such as high-pitch acquisition modes and advanced image reconstruction algorithms, have improved the calcification scores. These advances have reduced motion artifacts and improved consistency with ECG-gated results [40]. Non-ECG-gated CT is an alternative method that is less accurate than ECG-gated CT but still has a strong correlation. In routine clinical examinations, it is difficult to apply ECG gating to all CT examinations; therefore, we used non-ECG-gated CT. Third, 61 of the 200 patients underwent nCE-CCT using a different CT scanner on a different day from abdominal CTA, which may affect the Agatston scores due to different scanners, reconstruction methods, and kernels. We performed a subgroup analysis to determine whether there were any differences in the ability to differentiate CVD risk between the CT scanners used in nCE-CCT; however, the main scanner, the PCCT scanner, had better differentiation ability for PCV than for ACV, and the other CT scanner also had better specificity and accuracy for PCV than for ACV. There was no significant difference in diagnostic performance between ACV and PCV on the remaining two CT scanners, but PCV tended to have equal or better diagnostic performance. Therefore, it is believed that the difference in Agatston score measurements using different CT scanners has little effect on the diagnostic performance of PCV. However, the sample size for CT scanners other than the PCCT scanner was small; therefore, further studies with a larger number of cases are warranted.

Overall, ACV and PCV obtained using the PCCT angiography-based VNCa algorithm showed strong correlations with Agatston score. PCV showed significant differences across Agatston score grades. The performance in discriminating the risk of CVD events was significantly better for PCV than for ACV. The indices of 5.74 mL for ACV and 14.81% for PCV can effectively discriminate the risk of CVD events with high diagnostic accuracy and serve as clinically significant parameters. In particular, PCV serves as a crucial imaging biomarker for distinguishing high-risk CVD patients, providing a quantitative metric that enhances risk stratification and supports clinical decision-making.

## Supplementary Information

Below is the link to the electronic supplementary material.

Supplementary file1 (DOCX 18 KB)
