# Supplementary material for: Quantification of abdominal aortic calcification using photon-counting CT angiography: an imaging biomarker for high-risk cardiovascular patients
Source: Radiol Med. 2025 Mar 28;130(6):817–29. doi: 10.1007/s11547-025-01978-0 (PMC12185644; doi:10.1007/s11547-025-01978-0)
Supplement: Supplementary file 1 — Supplementary file1 (DOCX 18 KB) [file 11547_2025_1978_MOESM1_ESM.docx]

**Supplemental Table: Subgroup analysis to differentiate the risk of CVD events according to differences in CT scanners**

|  | **ACV (mL)** | **PCV (%)** | ***p*-value** |
| --- | --- | --- | --- |
| **NAEOTOM Alpha (Siemens)** | | | |
| **AUC (95% C.I.)** | 0.89 (0.82, 0.94) | 0.94 (0.88, 0.97) | **0.0002*** |
| **Sensitivity** | 73.1% (19/26) | 73.1% (19/26) | 1.00 |
| **Specificity** | 89.4% (101/113) | 100% (113/113) | N/A |
| **Accuracy** | 86.3% (120/139) | 95.0% (132/139) | **0.0005*** |
| **Aquilion Precision (Canon)** | | | |
| **AUC (95% C.I.)** | 0.93 (0.74, 0.99) | 0.95 (0.75, 0.99) | 0.75 |
| **Sensitivity** | 100% (7/7) | 85.7% (6/7) | N/A |
| **Specificity** | 66.7% (10/15) | 93.3% (14/15) | **0.046*** |
| **Accuracy** | 77.3% (17/22) | 90.9% (20/22) | **0.025*** |
| **Revolution CT (GE Healthcare)** | | | |
| **AUC (95% C.I.)** | 0.79 (0.79, 0.79) | 0.86 (0.86, 0.86) | N/A |
| **Sensitivity** | 100% (1/1) | 100% (1/1) | N/A |
| **Specificity** | 100% (14/14) | 100% (14/14) | N/A |
| **Accuracy** | 100% (15/15) | 100% (15/15) | N/A |
| **Aquilion One (Canon)** | | | |
| **AUC (95% C.I.)** | 0.89 (0.44, 0.99) | 0.89 (0.44, 0.99) | N/A |
| **Sensitivity** | 66.7% (2/3) | 66.7% (2/3) | 1.00 |
| **Specificity** | 95.2% (20/21) | 100% (21/21) | N/A |
| **Accuracy** | 91.7% (22/24) | 95.8% (23/24) | 0.32 |

ACV = aortic calcification volume; PCV = percentage calcification volume; AUC = area under the curve: 95% C.I. = 95% confidence interval

* The asterisks indicate statistically significant differences.
